# 4,4′-(Ethene-1,2-diyl)dipyridinium bis­[4-(2-carboxy­benzo­yl)benzoate]

**DOI:** 10.1107/S1600536809048417

**Published:** 2009-11-25

**Authors:** Cai Li, Dong-Sheng Li, Jun Zhao, Xue-Gang Zheng, Xi-Jun Ke

**Affiliations:** a College of Mechanical & Material Engineering, Functional Materials Research Institute, China Three Gorges University, Yichang 443002, People’s Republic of China; b Lanzhou Institute of Biological Products, Lanzhou 730046, People’s Republic of China

## Abstract

In the crystal structure of the title compound, C_12_H_12_N_2_^2+^·2C_15_H_9_O_5_^−^, the cation has site symmetry 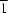 with the mid-point of C=C bond located on an inversion center. The two benzene rings of the anion are oriented at a dihedral angle 85.87 (6)°. In the crystal, inter­molecular O—H⋯O and N—H⋯O hydrogen bonds link the cations and anions into supra­molecular double chains, which are further connected into a three-dimensional network through inter­molecular C—H⋯O and π–π stacking between parallel pyridine rings [centroid–centroid distance = 3.4413 (12)Å] and between parallel benzene rings [centroid–centroid distance = 3.6116 (14)Å].

## Related literature

For hydrogen bonding and π–π stacking in supra­molecular systems, see: Desiraju (2000 ▶); Ma *et al.* (2006 ▶); Dong *et al.* (2008 ▶); Huang & Qian (2005 ▶).
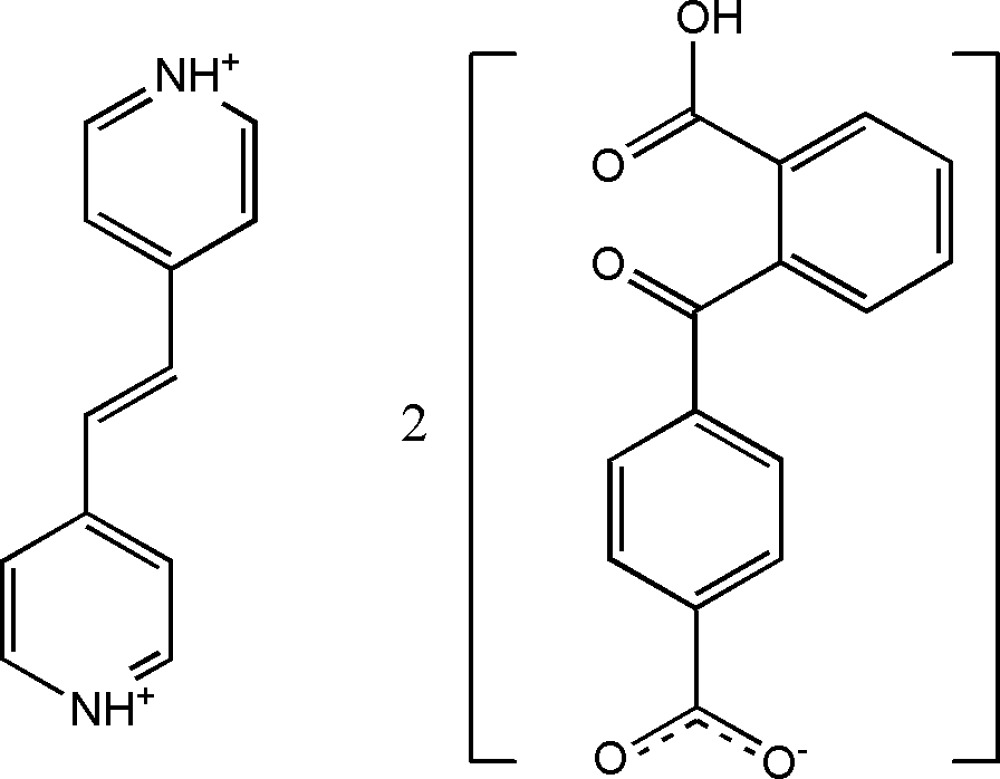


## Experimental

### 

#### Crystal data

C_12_H_12_N_2_^2+^·2C_15_H_9_O_5_^−^
*M* *_r_* = 722.68
Triclinic, 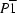

*a* = 7.1335 (13) Å
*b* = 9.4558 (17) Å
*c* = 13.206 (2) Å
α = 81.641 (2)°
β = 79.986 (2)°
γ = 71.260 (2)°
*V* = 826.9 (3) Å^3^
*Z* = 1
Mo *K*α radiation
μ = 0.11 mm^−1^
*T* = 293 K
0.29 × 0.14 × 0.06 mm

#### Data collection

Bruker SMART CCD diffractometer
Absorption correction: multi-scan (*SADABS*; Sheldrick, 1996 ▶) *T*_min_ = 0.983, *T*_max_ = 0.994
6357 measured reflections
3055 independent reflections
2212 reflections with *I* > 2σ(*I*)
*R*_int_ = 0.025

#### Refinement

*R*[*F* ^2^ > 2σ(*F* ^2^)] = 0.043
*wR*(*F* ^2^) = 0.115
*S* = 1.01
3055 reflections
250 parameters
2 restraints
H atoms treated by a mixture of independent and constrained refinement
Δρ_max_ = 0.20 e Å^−3^
Δρ_min_ = −0.22 e Å^−3^

### 

Data collection: *SMART* (Bruker, 1997 ▶); cell refinement: *SAINT* (Bruker, 1997 ▶); data reduction: *SAINT*; program(s) used to solve structure: *SHELXTL* (Sheldrick, 2008 ▶); program(s) used to refine structure: *SHELXTL*; molecular graphics: *SHELXTL*; software used to prepare material for publication: *SHELXTL*.

## Supplementary Material

Crystal structure: contains datablocks I, global. DOI: 10.1107/S1600536809048417/xu2637sup1.cif

Structure factors: contains datablocks I. DOI: 10.1107/S1600536809048417/xu2637Isup2.hkl

Additional supplementary materials:  crystallographic information; 3D view; checkCIF report

## Figures and Tables

**Table 1:** Hydrogen-bond geometry (Å, °)

*D*—H⋯*A*	*D*—H	H⋯*A*	*D*⋯*A*	*D*—H⋯*A*
N1—H1*A*⋯O1^i^	0.924 (17)	1.654 (17)	2.577 (2)	176 (2)
O5—H5⋯O2^ii^	0.91 (2)	1.72 (2)	2.629 (2)	175 (2)
C1—H1⋯O3^iii^	0.93	2.38	3.209 (3)	149
C5—H5*A*⋯O2^i^	0.93	2.46	3.144 (3)	130
C6—H6⋯O4^iv^	0.93	2.36	3.256 (2)	163

Symmetry codes: (i) 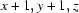; (ii) 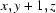; (iii) 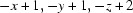; (iv) 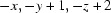.

**Fig. 1.**
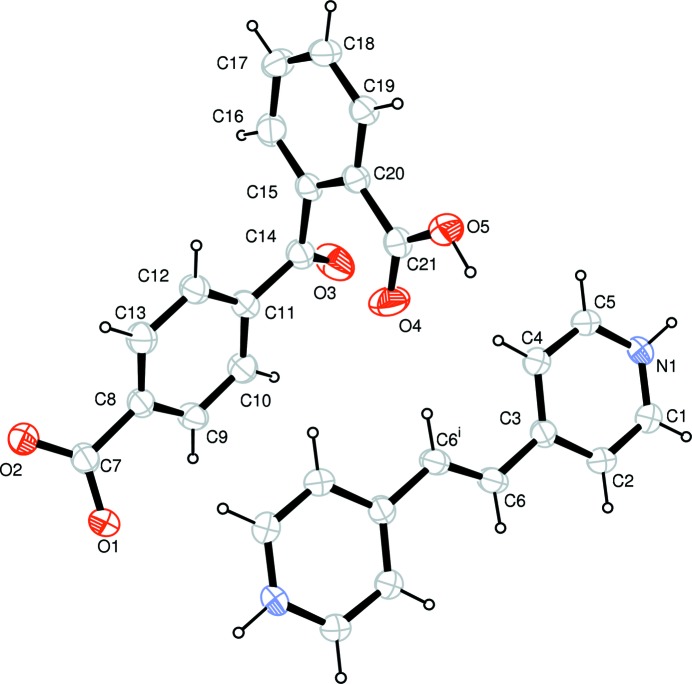
The structure of (I) with the atom-numbering scheme showing displacement ellipsoids at the 50% probability level [symmetry code: (i) -x, 1-y, 2-z].

**Fig. 2.**
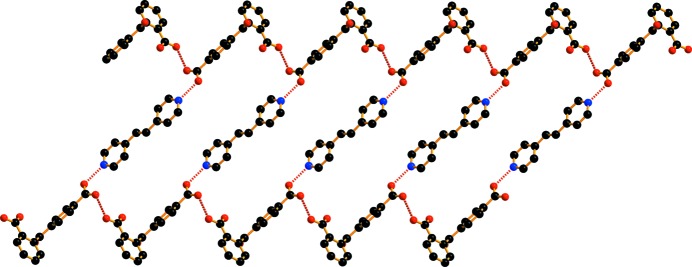
One-dimensional double chain connected by hydrogen bonds in the title complex.

**Fig. 3.**
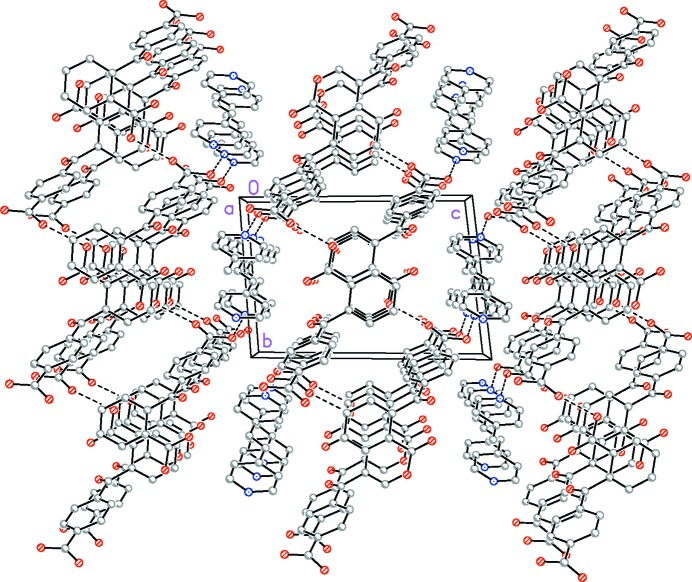
Supramolecular network formed by hydrogen-bonding and π-π stacking ineractions.

## supplementary crystallographic information

### Comment

Crystal structure formation depends on many factors such as temperature, solvent, nature of ligands and metals among many others, hydrogen bonding is one of the dominant factors due to the fact that they are relatively strong, directional, and able to act in concert with each other. Organic supramolecular systems have been well documented in the literature( Desiraju, 2000; Ma *et al.*, 2006; Dong *et al.*., 2008; Huang *et al.*, 2005). We attempted to synthesize a Zn^II^complex with the mixed ligand in hydrothermal synthesis conditions. However the title organic salt was obtained, its structure is reported here.

The asymmetric unit comprises one 4-(2-carboxybenzoyl)benzoate anion and half of diprotonated 4,4'-ethylenebis(pyridinium) cation (Fig 1). The dihedral angle between the two benzene rings of the anion is 85.87 (6)°, while the COOH(O4—C21—O5) group is co-planar with the phenyl ring and the deprotonated carboxylate COO(O1—C7—O2) group is slightly twisted with an angle of 31.93 (11)°. Intermolecular O—H···O and N—H···O hydrogen bonding links the ions into the supra-molecular double chains (Fig. 2). Furthermore, the double chains are stabilized by several distinct weak interactions which result in a three-dimensional supramolecular network: (*a*) π-π aromatic stacking between parallel pyridine rings [centroids distance = 3.4413 (12) Å]; (*b*) π-π aromatic stacking between parallel C15-benzene rings [centroids distance 3.6116 (14) Å]; (*c*) weak C—H···O hydrogen bonding (Table 1).

### Experimental

All chemicals were of reagent grade quality obtained from commercial sources and used without further purification. Zn(NO_3_)_2_.6H_2_O (0.0297 g, 0.1 mmol), 4,4'-ethylene-bis(pyridine) (0.0091 g, 0.05 mmol), 4-(2-carboxybenzoyl)benzoic acid (0.0270 g, 0.1 mmol) and water (15 ml) were placed in a 25 ml Teflon-lined stainless steel reactor and heated at 393 K for three days, and then cooled slowly to 298 K, at which time colourless crystals were obtained. The crystal used for data collection was obtained directly from the reaction mixture on cooling without further re-crystallization.

### Refinement

The H atoms bonded to C atoms were positioned geometrically (C—H = 0.93 Å) and allowed to ride on their parent atoms, with *U*_iso_(H) value equal to 1.2*U*_eq_(C). The H atoms bonded to O and N atoms were located in a difference Fourier map and refined with O—H distance restraint of 0.90±0.02 Å and N—H distance restraint of 0.93±0.02 Å, *U*_iso_(H) = 1.5*U*_eq_(O,N).

### Crystal data

**Table:**

C_12_H_12_N_2_^2+^·2C_15_H_9_O_5_^−^	*Z* = 1
*M**_r_* = 722.68	*F*(000) = 376
Triclinic, *P*1	*D*_x_ = 1.451 Mg m^−^^3^
Hall symbol: -P 1	Mo *K*α radiation, λ = 0.71073 Å
*a* = 7.1335 (13) Å	Cell parameters from 6357 reflections
*b* = 9.4558 (17) Å	θ = 2.7–25.5°
*c* = 13.206 (2) Å	µ = 0.11 mm^−^^1^
α = 81.641 (2)°	*T* = 293 K
β = 79.986 (2)°	Prism, colorless
γ = 71.260 (2)°	0.29 × 0.14 × 0.06 mm
*V* = 826.9 (3) Å^3^	

### Data collection

**Table:**

Bruker SMART CCD diffractometer	3055 independent reflections
Radiation source: fine-focus sealed tube	2212 reflections with *I* > 2σ(*I*)
graphite	*R*_int_ = 0.025
φ and ω scans	θ_max_ = 25.5°, θ_min_ = 2.7°
Absorption correction: multi-scan (*SADABS*; Sheldrick, 1996)	*h* = −8→8
*T*_min_ = 0.983, *T*_max_ = 0.994	*k* = −11→11
6357 measured reflections	*l* = −15→15

### Refinement

**Table:**

Refinement on *F*^2^	Primary atom site location: structure-invariant direct methods
Least-squares matrix: full	Secondary atom site location: difference Fourier map
*R*[*F*^2^ > 2σ(*F*^2^)] = 0.043	Hydrogen site location: inferred from neighbouring sites
*wR*(*F*^2^) = 0.115	H atoms treated by a mixture of independent and constrained refinement
*S* = 1.01	*w* = 1/[σ^2^(*F*_o_^2^) + (0.0527*P*)^2^ + 0.1979*P*] where *P* = (*F*_o_^2^ + 2*F*_c_^2^)/3
3055 reflections	(Δ/σ)_max_ < 0.001
250 parameters	Δρ_max_ = 0.20 e Å^−^^3^
2 restraints	Δρ_min_ = −0.22 e Å^−^^3^

### Special details

**Table:**

Geometry. All e.s.d.'s (except the e.s.d. in the dihedral angle between two l.s. planes) are estimated using the full covariance matrix. The cell e.s.d.'s are taken into account individually in the estimation of e.s.d.'s in distances, angles and torsion angles; correlations between e.s.d.'s in cell parameters are only used when they are defined by crystal symmetry. An approximate (isotropic) treatment of cell e.s.d.'s is used for estimating e.s.d.'s involving l.s. planes.
Refinement. Refinement of *F*^2^ against ALL reflections. The weighted *R*-factor *wR* and goodness of fit *S* are based on *F*^2^, conventional *R*-factors *R* are based on *F*, with *F* set to zero for negative *F*^2^. The threshold expression of *F*^2^ > σ(*F*^2^) is used only for calculating *R*-factors(gt) *etc*. and is not relevant to the choice of reflections for refinement. *R*-factors based on *F*^2^ are statistically about twice as large as those based on *F*, and *R*- factors based on ALL data will be even larger.

### Fractional atomic coordinates and isotropic or equivalent isotropic displacement parameters (Å^2^)

**Table:**

	*x*	*y*	*z*	*U*_iso_*/*U*_eq_	
C1	0.3708 (3)	0.7481 (2)	1.06116 (15)	0.0344 (5)	
H1	0.3941	0.7967	1.1118	0.041*	
C2	0.2259 (3)	0.6772 (2)	1.08332 (15)	0.0336 (5)	
H2	0.1539	0.6765	1.1492	0.040*	
C3	0.1860 (3)	0.6061 (2)	1.00748 (14)	0.0304 (4)	
C4	0.3013 (3)	0.6087 (2)	0.91086 (15)	0.0340 (5)	
H4	0.2803	0.5625	0.8581	0.041*	
C5	0.4460 (3)	0.6796 (2)	0.89372 (15)	0.0344 (5)	
H5A	0.5229	0.6799	0.8292	0.041*	
C6	0.0296 (3)	0.5327 (2)	1.03236 (15)	0.0349 (5)	
H6	−0.0340	0.5320	1.1001	0.042*	
C7	−0.1807 (3)	−0.1223 (2)	0.82078 (16)	0.0358 (5)	
C8	−0.0337 (3)	−0.0357 (2)	0.78080 (15)	0.0312 (4)	
C9	0.0829 (3)	−0.0123 (2)	0.84717 (15)	0.0347 (5)	
H9	0.0702	−0.0505	0.9161	0.042*	
C10	0.2171 (3)	0.0670 (2)	0.81169 (15)	0.0346 (5)	
H10	0.2976	0.0787	0.8562	0.042*	
C11	0.2329 (3)	0.1293 (2)	0.70999 (15)	0.0308 (4)	
C12	0.1136 (3)	0.1081 (2)	0.64353 (15)	0.0348 (5)	
H12	0.1210	0.1508	0.5755	0.042*	
C13	−0.0158 (3)	0.0235 (2)	0.67888 (16)	0.0372 (5)	
H13	−0.0910	0.0066	0.6336	0.045*	
C14	0.3742 (3)	0.2184 (2)	0.67376 (15)	0.0344 (5)	
C15	0.3715 (3)	0.3001 (2)	0.56687 (14)	0.0308 (4)	
C16	0.4987 (3)	0.2238 (2)	0.48697 (16)	0.0392 (5)	
H16	0.5756	0.1247	0.5004	0.047*	
C17	0.5124 (3)	0.2935 (2)	0.38745 (16)	0.0407 (5)	
H17	0.5966	0.2409	0.3343	0.049*	
C18	0.4011 (3)	0.4412 (2)	0.36712 (15)	0.0388 (5)	
H18	0.4115	0.4885	0.3004	0.047*	
C19	0.2744 (3)	0.5186 (2)	0.44582 (14)	0.0333 (5)	
H19	0.2000	0.6181	0.4318	0.040*	
C20	0.2565 (3)	0.4497 (2)	0.54578 (14)	0.0306 (4)	
C21	0.1207 (3)	0.5313 (2)	0.63185 (16)	0.0351 (5)	
N1	0.4789 (2)	0.74805 (18)	0.96750 (13)	0.0323 (4)	
H1A	0.575 (2)	0.797 (2)	0.9512 (16)	0.048*	
O1	−0.2576 (2)	−0.11219 (16)	0.91455 (11)	0.0438 (4)	
O2	−0.2190 (2)	−0.19672 (18)	0.76131 (12)	0.0511 (4)	
O3	0.5013 (2)	0.21679 (19)	0.72479 (12)	0.0567 (5)	
O4	0.1007 (3)	0.47083 (18)	0.71866 (11)	0.0581 (5)	
O5	0.0256 (2)	0.67303 (16)	0.60536 (11)	0.0434 (4)	
H5	−0.053 (3)	0.715 (3)	0.6618 (13)	0.065*	

### Atomic displacement parameters (Å^2^)

**Table:**

	*U*^11^	*U*^22^	*U*^33^	*U*^12^	*U*^13^	*U*^23^
C1	0.0388 (11)	0.0335 (11)	0.0339 (11)	−0.0137 (9)	−0.0039 (9)	−0.0075 (9)
C2	0.0361 (11)	0.0372 (11)	0.0282 (11)	−0.0141 (9)	0.0014 (8)	−0.0052 (9)
C3	0.0339 (11)	0.0290 (10)	0.0304 (11)	−0.0128 (8)	−0.0044 (8)	−0.0019 (8)
C4	0.0387 (11)	0.0370 (11)	0.0306 (11)	−0.0172 (9)	−0.0019 (9)	−0.0069 (9)
C5	0.0389 (11)	0.0386 (11)	0.0276 (11)	−0.0166 (9)	0.0011 (9)	−0.0045 (9)
C6	0.0382 (11)	0.0430 (12)	0.0274 (11)	−0.0212 (9)	0.0008 (8)	−0.0017 (9)
C7	0.0335 (11)	0.0321 (11)	0.0429 (13)	−0.0137 (9)	0.0023 (9)	−0.0080 (9)
C8	0.0278 (10)	0.0265 (10)	0.0377 (11)	−0.0080 (8)	0.0014 (8)	−0.0059 (8)
C9	0.0391 (11)	0.0334 (11)	0.0302 (11)	−0.0133 (9)	−0.0009 (9)	0.0017 (9)
C10	0.0362 (11)	0.0371 (11)	0.0328 (11)	−0.0134 (9)	−0.0081 (9)	−0.0014 (9)
C11	0.0311 (10)	0.0290 (10)	0.0324 (11)	−0.0108 (8)	−0.0033 (8)	−0.0005 (8)
C12	0.0380 (11)	0.0372 (11)	0.0312 (11)	−0.0158 (9)	−0.0046 (9)	−0.0002 (9)
C13	0.0370 (11)	0.0426 (12)	0.0367 (12)	−0.0172 (10)	−0.0068 (9)	−0.0047 (9)
C14	0.0370 (11)	0.0361 (11)	0.0334 (11)	−0.0157 (9)	−0.0055 (9)	−0.0021 (9)
C15	0.0334 (10)	0.0349 (11)	0.0300 (11)	−0.0193 (9)	−0.0035 (8)	−0.0024 (8)
C16	0.0413 (12)	0.0354 (11)	0.0429 (13)	−0.0138 (10)	−0.0047 (10)	−0.0064 (10)
C17	0.0422 (12)	0.0490 (13)	0.0324 (12)	−0.0173 (10)	0.0045 (9)	−0.0120 (10)
C18	0.0471 (12)	0.0481 (13)	0.0261 (11)	−0.0237 (11)	−0.0008 (9)	−0.0024 (9)
C19	0.0371 (11)	0.0365 (11)	0.0306 (11)	−0.0181 (9)	−0.0029 (9)	−0.0027 (9)
C20	0.0303 (10)	0.0372 (11)	0.0298 (10)	−0.0179 (9)	−0.0019 (8)	−0.0051 (8)
C21	0.0390 (11)	0.0384 (12)	0.0344 (12)	−0.0208 (10)	−0.0029 (9)	−0.0059 (9)
N1	0.0332 (9)	0.0313 (9)	0.0361 (9)	−0.0170 (7)	−0.0019 (7)	−0.0020 (7)
O1	0.0517 (9)	0.0507 (9)	0.0365 (8)	−0.0315 (8)	0.0090 (7)	−0.0094 (7)
O2	0.0518 (10)	0.0595 (10)	0.0518 (10)	−0.0337 (8)	0.0162 (7)	−0.0255 (8)
O3	0.0625 (11)	0.0779 (12)	0.0471 (10)	−0.0449 (10)	−0.0238 (8)	0.0134 (8)
O4	0.0851 (13)	0.0520 (10)	0.0288 (9)	−0.0186 (9)	0.0082 (8)	−0.0017 (7)
O5	0.0459 (9)	0.0397 (9)	0.0406 (9)	−0.0121 (7)	0.0067 (7)	−0.0084 (7)

### Geometric parameters (Å, °)

**Table:**

C1—N1	1.339 (2)	C11—C12	1.398 (3)
C1—C2	1.374 (3)	C11—C14	1.488 (3)
C1—H1	0.9300	C12—C13	1.388 (3)
C2—C3	1.398 (3)	C12—H12	0.9300
C2—H2	0.9300	C13—H13	0.9300
C3—C4	1.394 (3)	C14—O3	1.215 (2)
C3—C6	1.462 (3)	C14—C15	1.508 (3)
C4—C5	1.373 (3)	C15—C16	1.387 (3)
C4—H4	0.9300	C15—C20	1.404 (3)
C5—N1	1.336 (2)	C16—C17	1.384 (3)
C5—H5A	0.9300	C16—H16	0.9300
C6—C6^i^	1.318 (4)	C17—C18	1.380 (3)
C6—H6	0.9300	C17—H17	0.9300
C7—O2	1.244 (2)	C18—C19	1.379 (3)
C7—O1	1.268 (2)	C18—H18	0.9300
C7—C8	1.511 (3)	C19—C20	1.388 (3)
C8—C13	1.381 (3)	C19—H19	0.9300
C8—C9	1.392 (3)	C20—C21	1.489 (3)
C9—C10	1.379 (3)	C21—O4	1.211 (2)
C9—H9	0.9300	C21—O5	1.320 (2)
C10—C11	1.387 (3)	N1—H1A	0.924 (17)
C10—H10	0.9300	O5—H5	0.91 (2)
			
N1—C1—C2	120.83 (18)	C13—C12—H12	119.9
N1—C1—H1	119.6	C11—C12—H12	119.9
C2—C1—H1	119.6	C8—C13—C12	120.46 (19)
C1—C2—C3	120.38 (18)	C8—C13—H13	119.8
C1—C2—H2	119.8	C12—C13—H13	119.8
C3—C2—H2	119.8	O3—C14—C11	121.52 (18)
C4—C3—C2	117.10 (17)	O3—C14—C15	119.46 (17)
C4—C3—C6	123.40 (17)	C11—C14—C15	118.76 (17)
C2—C3—C6	119.50 (17)	C16—C15—C20	119.20 (18)
C5—C4—C3	119.87 (18)	C16—C15—C14	117.15 (18)
C5—C4—H4	120.1	C20—C15—C14	123.56 (17)
C3—C4—H4	120.1	C17—C16—C15	120.7 (2)
N1—C5—C4	121.57 (18)	C17—C16—H16	119.7
N1—C5—H5A	119.2	C15—C16—H16	119.7
C4—C5—H5A	119.2	C18—C17—C16	120.02 (19)
C6^i^—C6—C3	126.0 (2)	C18—C17—H17	120.0
C6^i^—C6—H6	117.0	C16—C17—H17	120.0
C3—C6—H6	117.0	C19—C18—C17	119.91 (19)
O2—C7—O1	124.59 (18)	C19—C18—H18	120.0
O2—C7—C8	119.16 (18)	C17—C18—H18	120.0
O1—C7—C8	116.25 (18)	C18—C19—C20	120.85 (19)
C13—C8—C9	119.21 (18)	C18—C19—H19	119.6
C13—C8—C7	120.68 (18)	C20—C19—H19	119.6
C9—C8—C7	120.10 (18)	C19—C20—C15	119.32 (18)
C10—C9—C8	120.65 (18)	C19—C20—C21	121.69 (18)
C10—C9—H9	119.7	C15—C20—C21	118.99 (17)
C8—C9—H9	119.7	O4—C21—O5	123.48 (19)
C9—C10—C11	120.41 (19)	O4—C21—C20	122.0 (2)
C9—C10—H10	119.8	O5—C21—C20	114.51 (17)
C11—C10—H10	119.8	C5—N1—C1	120.23 (17)
C10—C11—C12	119.07 (17)	C5—N1—H1A	118.1 (14)
C10—C11—C14	119.72 (17)	C1—N1—H1A	121.6 (14)
C12—C11—C14	121.21 (17)	C21—O5—H5	109.1 (16)
C13—C12—C11	120.14 (18)		
			
N1—C1—C2—C3	−1.3 (3)	C10—C11—C14—C15	−172.89 (18)
C1—C2—C3—C4	1.1 (3)	C12—C11—C14—C15	6.5 (3)
C1—C2—C3—C6	−179.33 (18)	O3—C14—C15—C16	83.1 (3)
C2—C3—C4—C5	−0.2 (3)	C11—C14—C15—C16	−91.1 (2)
C6—C3—C4—C5	−179.75 (19)	O3—C14—C15—C20	−93.4 (2)
C3—C4—C5—N1	−0.6 (3)	C11—C14—C15—C20	92.4 (2)
C4—C3—C6—C6^i^	−3.6 (4)	C20—C15—C16—C17	−0.1 (3)
C2—C3—C6—C6^i^	176.9 (3)	C14—C15—C16—C17	−176.80 (18)
O2—C7—C8—C13	31.7 (3)	C15—C16—C17—C18	1.0 (3)
O1—C7—C8—C13	−147.9 (2)	C16—C17—C18—C19	−0.8 (3)
O2—C7—C8—C9	−149.50 (19)	C17—C18—C19—C20	−0.2 (3)
O1—C7—C8—C9	30.9 (3)	C18—C19—C20—C15	1.0 (3)
C13—C8—C9—C10	−1.0 (3)	C18—C19—C20—C21	179.95 (18)
C7—C8—C9—C10	−179.80 (18)	C16—C15—C20—C19	−0.8 (3)
C8—C9—C10—C11	2.3 (3)	C14—C15—C20—C19	175.59 (17)
C9—C10—C11—C12	−1.2 (3)	C16—C15—C20—C21	−179.81 (17)
C9—C10—C11—C14	178.19 (18)	C14—C15—C20—C21	−3.4 (3)
C10—C11—C12—C13	−1.1 (3)	C19—C20—C21—O4	178.18 (19)
C14—C11—C12—C13	179.44 (18)	C15—C20—C21—O4	−2.9 (3)
C9—C8—C13—C12	−1.4 (3)	C19—C20—C21—O5	−1.8 (3)
C7—C8—C13—C12	177.40 (18)	C15—C20—C21—O5	177.15 (16)
C11—C12—C13—C8	2.5 (3)	C4—C5—N1—C1	0.4 (3)
C10—C11—C14—O3	13.1 (3)	C2—C1—N1—C5	0.5 (3)
C12—C11—C14—O3	−167.5 (2)		

Symmetry codes: (i) −*x*, −*y*+1, −*z*+2.

### Hydrogen-bond geometry (Å, °)

**Table:**

*D*—H···*A*	*D*—H	H···*A*	*D*···*A*	*D*—H···*A*
N1—H1A···O1^ii^	0.92 (2)	1.65 (2)	2.577 (2)	176 (2)
O5—H5···O2^iii^	0.91 (2)	1.72 (2)	2.629 (2)	175 (2)
C1—H1···O3^iv^	0.93	2.38	3.209 (3)	149
C5—H5A···O2^ii^	0.93	2.46	3.144 (3)	130
C6—H6···O4^i^	0.93	2.36	3.256 (2)	163

Symmetry codes: (ii) *x*+1, *y*+1, *z*; (iii) *x*, *y*+1, *z*; (iv) −*x*+1, −*y*+1, −*z*+2; (i) −*x*, −*y*+1, −*z*+2.
